# Varicella Pneumonia in Immunocompetent Adults: Symptomatic and Asymptomatic Cases

**DOI:** 10.7759/cureus.68891

**Published:** 2024-09-07

**Authors:** Sarah Qasem, Rawan Almutairi, Mohammed Elhousiny, Abeer Albazzali

**Affiliations:** 1 Department of Dermatology, Amiri Hospital, Kuwait City, KWT; 2 Department of Dermatology, Farwaniya Hospital, Kuwait City, KWT; 3 Department of Infectious Diseases, Infectious Diseases Hospital, Kuwait City, KWT

**Keywords:** adult varicella, chickenpox, immunocompetent adult, infection related complications, radiographic screening, varicella pneumonia, varicella vaccines, varicella-zoster virus

## Abstract

Varicella zoster virus (VZV) is an enveloped, linear double-stranded DNA virus. It belongs to the Herpesviridae family and can manifest as primary varicella infection or secondary infection, also known as herpes zoster. Varicella pneumonia is an uncommon but potentially life-threatening complication of primary varicella infection. It mainly affects adults, and, if left untreated, the mortality rate is high.

We report two cases involving adult male patients who presented with a generalized widespread vesicular rash compatible with primary varicella. Each patient had a different clinical presentation; the first patient had respiratory symptoms, while the second patient did not. Chest radiographs of both patients showed bilateral infiltrates. Treatment was initiated with the administration of intravenous acyclovir with a very good response. This report of two cases highlights the importance of early detection and prompt treatment of varicella-related complications, especially in higher-risk patients, to reduce morbidity and mortality and improve overall clinical outcomes. We also aim to reinforce the importance of immunization, which would aid in reducing the incidence of vaccine-preventable diseases such as varicella and its life-threatening complications.

## Introduction

Initial infection with varicella zoster virus (VZV) causes primary varicella, also known as chickenpox. The course of primary varicella is usually mild in children; however, its complications were found to be approximately 25 times more severe in adults [1]. Varicella pneumonia is a rare but serious, life-threatening complication in adults with primary varicella, and acyclovir remains the mainstay of treatment. Early treatment with acyclovir is associated with decreased morbidity and mortality. Factors that increase the risk of varicella pneumonia include smoking, male gender, immunosuppression, and an older age of onset of primary varicella infection [2]. Physicians should maintain a high index of suspicion for varicella pneumonia in adult patients with primary VZV infection, especially those with the risk factors mentioned above. This will help reduce mortality and prevent long-term complications.

## Case presentation

Case A

**Figure 1:**
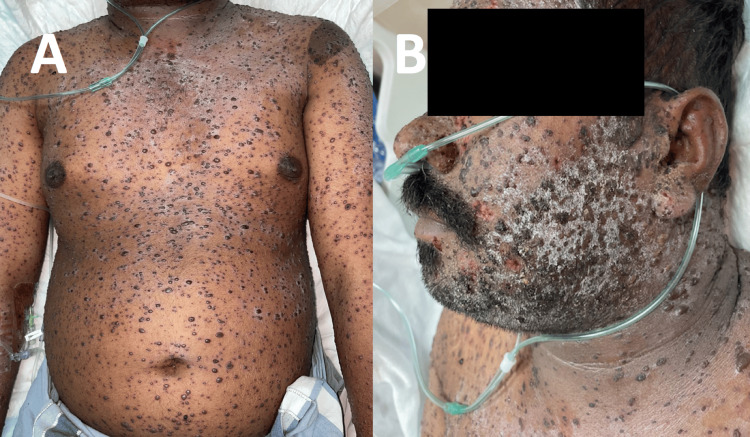
Case A pre-treatment images Widespread pleomorphic rash, consisting of papules, vesicles, and crusts seen on (A) trunk and upper extremities, (B) face and neck, which are extensively affected, with areas of bleeding lesions

A 34-year-old Indian male presented to the Infectious Diseases Hospital with a two-day history of an extensive painful vesicular rash covering most of the body (Figures 1A, 1B). The patient also reported a history of fever associated with chills one day after the appearance of the rash. He also complained of fatigue and decreased appetite. Two days after the rash onset, the patient was still febrile and started to complain of shortness of breath and palpitations, and he subsequently presented to the Emergency Department of the Infectious Diseases Hospital. He denied abdominal pain, nausea/vomiting, headaches, changes in vision or hearing, and abnormal body movements. His Past medical history was unremarkable and he reported that he had never had chickenpox; he had not received the varicella vaccine either. He had been a heavy smoker for 20 years.

In the ER, the patient's vital signs were as follows - body temperature: 39.6 °C, pulse rate: 130 beats/minute, respiratory rate: 24 breaths/minute, and blood pressure: 120/70 mmHg. Oxygen saturation measured by pulse oximetry showed a 93% oxygen saturation on room air. The skin inspection revealed widespread vesicles of different stages, and crusts were observed on an erythematous base, covering the face, neck, trunk, as well as upper and lower extremities. Auscultation of the chest revealed bilateral decreased air entry, as well as crepitation in the lower and middle lobes bilaterally. The rest of the physical examination was unremarkable. 

**Figure 2:**
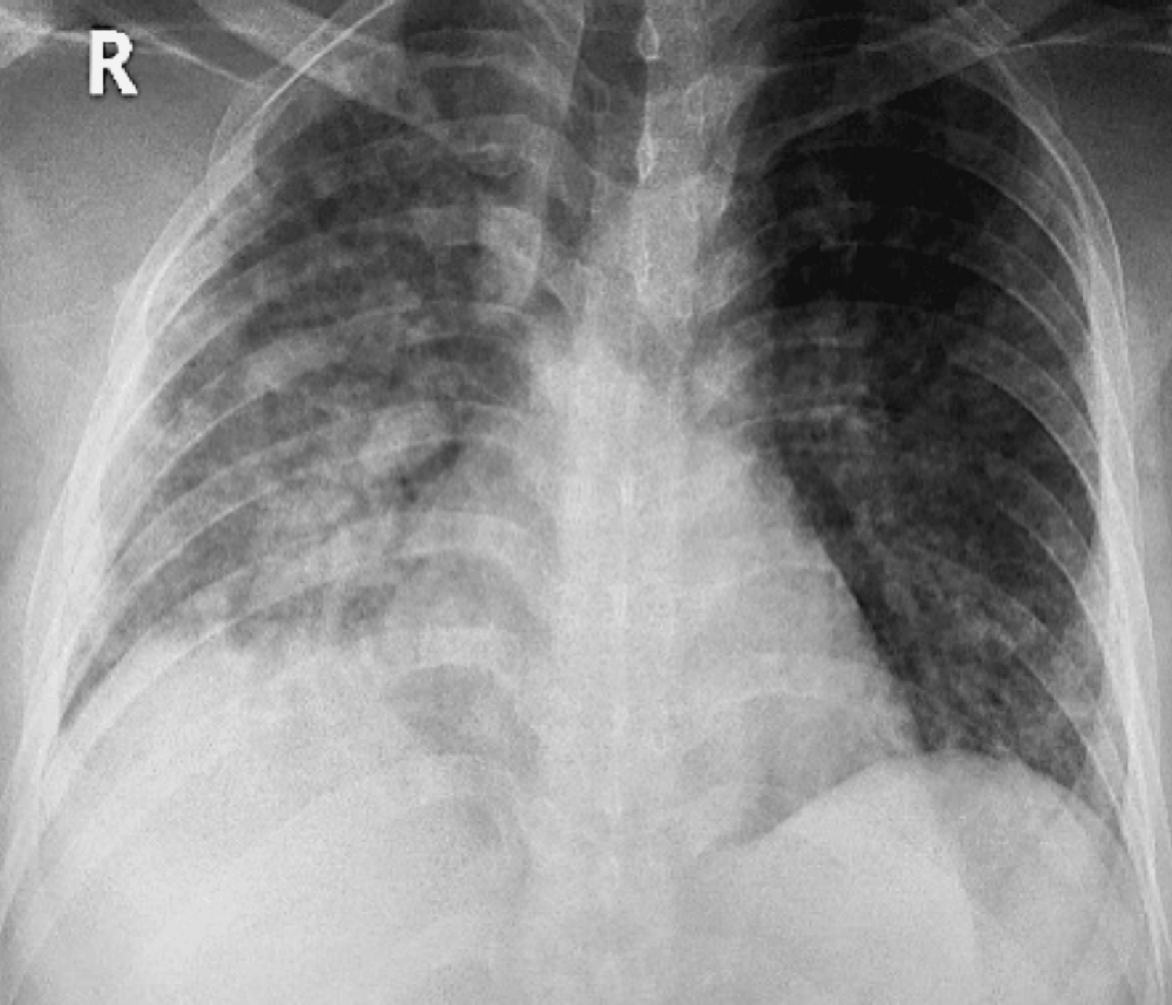
Chest radiograph of Case A on the day of presentation The image shows ground glass opacification with nodular infiltration seen in the right lung field

Laboratory tests revealed elevated total white blood cell count (12 x10^9^/L), mainly an increase in lymphocytes (Table 1). Arterial blood gas analysis, as shown in Table 1, was significant for respiratory alkalosis (pH: 7.49). The chemistry panel was unremarkable. Chest radiograph revealed ground glass opacification with infiltration bilaterally in all lung fields, more pronounced on the right side (Figure 2). 

**Table 1:** Complete blood count and arterial blood gas analysis of Case A pH: acidity/alkalinity; PO_2_: partial pressure of oxygen; PCO_2_: partial pressure of carbon dioxide; HCO_3_: bicarbonates; SO_2_: oxygen saturation

Laboratory parameters	Patient value	Reference range
Hemoglobin	155	130-180 g/L
White blood cell count	12	4.0-11.0 x10^9^/L
Lymphocytes	3.44	1.2-3.6 x10^9^/L
Platelets	190	150-450 x10^9^/L
Reticulocyte count	3.2%	0.5-2.5%
Red blood cell count	4.5	4.0-6.5 x10^12^/L
pH	7.49	7.35-7.45
PO_2_	90	80-100 mmHg
PCO_2_	27	35-45 mmHg
HCO_3_	18	22-26 mEq/L
SO_2_	92%	>95%

The patient was diagnosed with a case of chickenpox complicated with pneumonia and was managed immediately with intravenous (IV) fluids, paracetamol, and oxygen supplementation using a nasal cannula, and the oxygen saturation was maintained on 4 liters. In addition, he was placed on 650 mg acyclovir IV every eight hours and empirical 400 mg moxifloxacin IV once daily, both for five days. Also, the patient was started on 1 gram meropenem IV every eight hours for 10 days. Calamine lotion was applied on all skin lesions regularly.

In the ward, on the second day of admission, the patient reported improvement in terms of respiratory symptoms but was still febrile, and his oxygen saturation was 93%; he still required supplemental oxygen. After four days, his condition improved noticeably; he reported improvement in skin rash, denied respiratory symptoms, and was afebrile. He was gradually weaned off oxygen supplementation, and the pH range normalized as the respiratory rate returned to normal. Renal function tests were monitored regularly, and no deterioration was observed. Further investigations revealed non-reactive (negative) HIV, hepatitis B virus (HBV), and hepatitis C virus (HCV). The patient was subsequently discharged home after completing seven days of IV acyclovir and was instructed to continue taking it orally at home. At the one-week follow-up at the outpatient department, significant improvement was observed overall and the patient was satisfied with the results. 

Case B

**Figure 3:**
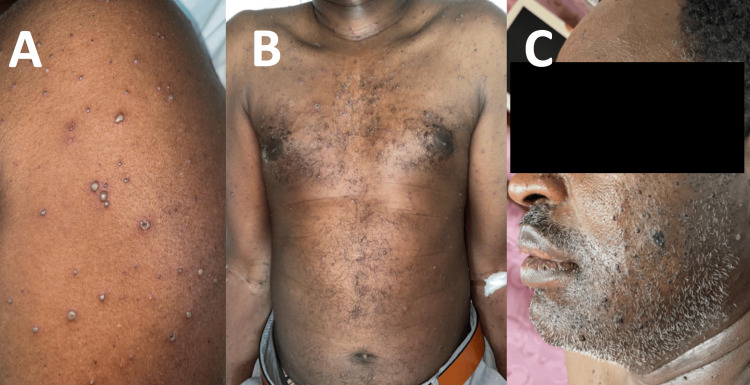
Case B pre-treatment images Vesicles, papules, pustules, and scabs are seen on (A) upper limbs, (B) trunk, and (C) face, mostly affecting the beard area

A 50-year-old Sudani male, with a known case of hypertension, presented to the Emergency Department with a widespread rash of two days' duration, accompanied by painful, itchy vesicles scattered throughout the trunk, upper and lower extremities, as well as the face (Figures 3A-3C). The patient also reported a fever for two days, concomitant with the appearance of skin lesions. The rash had started mild and increased in severity and pain over the past two days; the patient had sought medical attention at a primary care clinic and had been referred to the Emergency Department of the Infectious Diseases Hospital.

As for his past medical history, the patient reported that he had been diagnosed with hypertension four years ago, for which he takes amlodipine 5 mg oral tablets daily. He also gave a history of malaria diagnosis, three years ago back in his native country, Sudan, which had been treated successfully. He denied any contact with chickenpox patients. In addition, he denied experiencing chickenpox previously, and he stated that he had never received the varicella vaccine. 

On admission, the patient's vital signs were as follows - body temperature: 39 °C, pulse rate: 88 beats/minute, respiratory rate: 20 breaths/minute, and blood pressure: 125/85 mmHg. Pulse oximetry showed a 100% oxygen saturation on room air. On inspection of the skin, a diffuse pleomorphic vesicular rash, compatible with primary varicella, was seen on the face, neck, and trunk, as well as the upper and lower extremities. Chest auscultation revealed normal breath sounds and equal air entry bilaterally. The abdomen was soft and non-tender. The rest of the physical examination was unremarkable.

**Figure 4:**
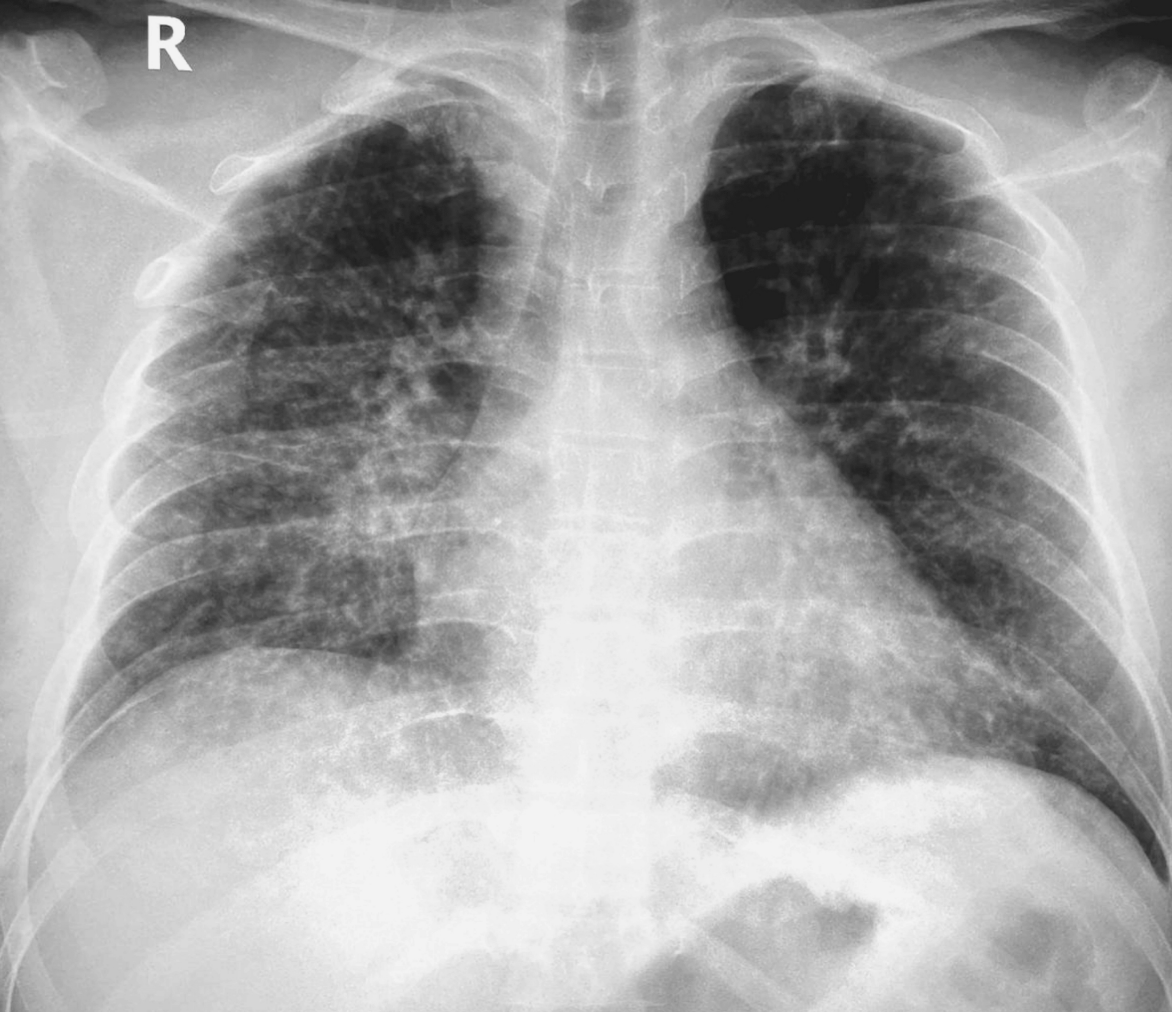
Chest radiograph of Case B The image shows ground glass opacities and infiltrations bilaterally

Laboratory investigations are shown in Table 2. There was an increase in total white blood cell count (11.3 x10^9^/L), mainly an increase in lymphocytes (4.2 x10^9^/L) and monocytes (2.5 x10^9^/L). In addition, as shown in Table 2, there was a derangement of liver enzymes: alanine transaminase (ALT) and aspartate aminotransferase (AST) were 127 U/L and 108 U/L, respectively. Chest radiograph revealed predominant ground glass opacities with nodular infiltrations bilaterally (Figure 4). A diagnosis of silent varicella pneumonia was made and the patient was managed promptly with IV fluids and paracetamol for pain management. In addition, he was started on a seven-day course of 500 mg acyclovir IV every eight hours, as well as 400 mg moxifloxacin orally once daily for five days. Cetirizine 10 mg oral tablets were prescribed to relieve itching and calamine lotion was applied and occluded on the skin.

**Table 2:** Complete blood count and chemistry profile laboratory findings of Case B ALP: alkaline phosphatase; ALT: alanine transaminase; AST: aspartate aminotransferase; GGT: gamma-glutamyl transferase; LDH: lactate dehydrogenase

Laboratory parameters	Patient value	Reference range
Hemoglobin	155	130-180 g/L
White blood cell count	11.3	4-11 x10^9^/L
Lymphocytes	4.2	1.2-3.6 x10^9^/L
Monocytes	2.5	0.3-0.9 x10^9^/L
Platelets	167	150-450 x10^9^/L
Reticulocyte count	3.3%	0.5-2.5%
Red blood cell count	5.37	4.5-6.5 x10^12^/L
Glucose	8.49	4.1-5.6 mmol/L
Creatinine	92	64-104 umol/L
Direct bilirubin	3.5	0.5-3.4 umol/L
Indirect bilirubin	12	1-20 umol/L
Total bilirubin	15.5	0-21 umol/L
ALT	127	30-50 U/L
AST	108	30-50 U/L
ALP	103	43-115 U/L
LDH	704	4-248 U/L
GGT	146	2-55 U/L

On the second day of admission, the patient developed two spikes of fever and was managed accordingly with paracetamol. No new complaints or respiratory symptoms were reported during the patient's in-patient course, and skin lesions began to crust. Renal function tests were monitored regularly with no deterioration. Virology laboratory investigations of HIV, HBV, and HCV were all non-reactive (negative).

The patient was then discharged home after completing a seven-day course of IV acyclovir, and he was prescribed acyclovir per oral to be continued at home as instructed. After one week, the patient presented to the outpatient clinic for his follow-up appointment and reported a noticeable improvement in his skin rash; he was symptom-free and satisfied with the results.

## Discussion

VZV is a highly contagious virus, and its transmission most commonly occurs by an airborne route. The other ways of virus transmission include direct contact with an infected vesicle, conjunctival fluid, or saliva of an infected patient [3]. The virus may enter through the respiratory system or conjunctiva of the host [1]. Transmission can occur approximately two days before the outbreak of rash, and there is no longer a risk of infectivity by direct touch when vesicular lesions have crusted.

In primary varicella infection, VZV invades the upper respiratory tract mucosa and gains access to regional lymph nodes, where it replicates and infects T-cells, mainly memory T-cells, resulting in primary viremia within four to six days of infection. Infected T-cells then travel in the blood and exit the bloodstream via endothelial cells of skin capillaries, reaching the epidermal cells. After an incubation period of 10-21 days, vesicular exanthematous eruption appears [4]. In primary infection, the virus travels retrogradely by neuronal axons or hematogenously by infected T-cells until it reaches the dorsal root ganglia of the spine, where it remains latent for life, until reactivation, which can happen decades later, either spontaneously or in response to a variety of trigger factors that decrease cell-mediated immunity of infected patients, such as steroids use, malignancy, and diabetes mellitus [5]. Also, varicella reactivation increases with advancing age. Reactivation of VZV results in secondary infection, where the virus, in contrast with primary infection, travels anterogradely from the dorsal root ganglia to the skin nerve terminals, causing pain and skin rash. Secondary infection is also known as herpes zoster (shingles) and is marked by a dermatomal distribution of pruritic cutaneous vesicular lesions [6,7]. Chickenpox and shingles have similar morphology of vesicular lesions but the distribution on the body varies.

The introduction of the varicella vaccine in 1995 led to a major reduction in varicella incidence in all ages, and after implementing a second dose in 2006, the incidence fell further [8]. Despite the significant drop in the incidence of varicella, hospitalizations and deaths due to varicella-related complications are reported every year, in patients of all ages. The vast majority of reported varicella complications and mortality occur in immunocompromised, pregnant, and adult patients. Primary varicella can lead to viral complications like herpes-zoster cerebellitis and encephalitis, as well as bacterial complications like pneumonia and cellulitis [9]. Varicella pneumonia is a rare but serious complication and it affects approximately one out of every 400 adults with chickenpox [1]. Smoking, pregnancy, immunosuppression, and male gender are considered risk factors for varicella pneumonia [2]. 

As previously stated, pulmonary involvement occurs in 5-15% of adult cases of chickenpox, and respiratory symptoms are typically progressive and occur within one to six days of cutaneous eruption. Varicella pneumonia and hospitalization due to varicella infection seem to more commonly affect adults [10]. Respiratory involvement often presents as dry cough, shortness of breath, and tachypnea. Chest radiograph findings suggestive of varicella pneumonia include pulmonary nodules with surrounding bilateral ground glass opacification. After the healing of skin lesions, radiological findings usually resolve as well [11]. 

The first-line treatment of complicated or severe varicella infection is acyclovir, and depending on the case and severity of the infection, oral or intravenous acyclovir is given. Immunocompromised patients should be treated with intravenous acyclovir, due to the reduced bioavailability when given orally. Both liver and kidney functions should be monitored in patients treated with acyclovir, and nephrotoxic drugs are contraindicated during this course. If serious VZV infection is present in patients who are acyclovir-resistant, treatment with foscarnet is advised.

## Conclusions

Varicella pneumonia is a rare but life-threatening complication of primary varicella infection. It is more common and severe in adults. The incidence of primary varicella infection has dropped significantly after the introduction of the varicella vaccine but varicella-related hospitalizations and deaths are still reported. Complications are common in the adult age group, as well as in pregnant and immunocompromised individuals. We discussed two cases of primary varicella infection, both symptomatic and asymptomatic, complicated with varicella pneumonia evident on chest radiographs. Both patients were treated successfully with IV acyclovir and were symptom-free at their one-week outpatient follow-up. This report highlights the importance of high clinical suspicion, early detection, and prompt treatment of varicella-related complications to improve long-term clinical outcomes in these patients.
